# Phenotypic and genetic aspects of hereditary ataxia in dogs

**DOI:** 10.1111/jvim.16742

**Published:** 2023-06-21

**Authors:** Kimberley Stee, Mario Van Poucke, Mark Lowrie, Luc Van Ham, Luc Peelman, Natasha Olby, Sofie F.M. Bhatti

**Affiliations:** ^1^ Small Animal Department Faculty of Veterinary Medicine, Ghent University Merelbeke Belgium; ^2^ Department of Veterinary and Biosciences Faculty of Veterinary Sciences, Ghent University Merelbeke Belgium; ^3^ Dovecote Veterinary Hospital Derby UK; ^4^ Department of Clinical Sciences North Carolina State University Raleigh North Carolina USA

**Keywords:** (spino)cerebellar, canine, gene, genetic variant, multiple system degeneration, neuronal ceroid lipofuscinosis

## Abstract

Hereditary ataxias are a large group of neurodegenerative diseases that have cerebellar or spinocerebellar dysfunction as core feature, occurring as an isolated sign or as part of a syndrome. Based on neuropathology, this group of diseases has so far been classified into cerebellar cortical degenerations, spinocerebellar degenerations, cerebellar ataxias without substantial neurodegeneration, canine multiple system degeneration, and episodic ataxia. Several new hereditary ataxia syndromes are described, but most of these diseases have similar clinical signs and unspecific diagnostic findings, wherefore achieving a definitive diagnosis in these dogs is challenging. Eighteen new genetic variants associated with these diseases have been discovered in the last decade, allowing clinicians to reach a definitive diagnosis for most of these conditions, and allowing breeding schemes to adapt to prevent breeding of affected puppies. This review summarizes the current knowledge about hereditary ataxias in dogs, and proposes to add a “multifocal degenerations with predominant (spino)cerebellar component” category regrouping canine multiple system degeneration, new hereditary ataxia syndromes that do not fit in 1 of the previous categories, as well as specific neuroaxonal dystrophies and lysosomal storage diseases that cause major (spino)cerebellar dysfunction.

**Abbreviations**
ACMG: American College of Medical Genetics and Genomics
ARSG: arylsulfatase G
ATG4D: autophagy‐related 4D cysteine peptidase
ATP1B2: ATPase Na^+^/K^+^ transporting subunit beta 2
CAPN1: calpain 1
CCD: cerebellar cortical degeneration
CDMC: cerebellar degeneration‐myositis complex
CFA: dog chromosome
CK: creatine kinase
CMSD: canine multiple system degeneration
CNS: central nervous system
CTSD: cathepsin D
delins: deletion‐insertion sequence change
GLB1: galactosidase beta 1
GRM1: glutamate metabotropic receptor 1
HACE1: HECT domain and ankyrin repeat containing E3 ubiquitin protein ligase 1
HE: hematoxylin‐eosin
ITPR1: inositol 1,4,5‐triphosphate receptor type 1
KCNIP4: potassium voltage‐gated channel interacting protein 4
KCNJ10: potassium inwardly rectifying channel subfamily J member 10
LFB: Luxol fast blue
LOA: late‐onset ataxia
MRI: magnetic resonance imaging
NAD: neuroaxonal dystrophy
NCL: neuronal ceroid lipofuscinosis
PNPLA8: patatin‐like phospholipase domain containing 8
PNS: peripheral nervous system
RAB24: RAB24, member RAS oncogene family
SAMS: spinocerebellar ataxia with (or without) myokymia, seizures, or both
SCA: spinocerebellar ataxia
SCN8A: sodium voltage‐gated channel alpha subunit 8
SDCA: spongy degeneration and cerebellar ataxia
SEL1L: SEL1L adaptor subunit of ERAD E3 ubiquitin ligase
SELENOP: selenoprotein P
SERAC1: serine active site containing 1
SINE: short interspersed nuclear element
SLC12A6: solute carrier family 12 member 6
SLC25A12: solute carrier family 25 member 12
SNX14: sorting nexin 14
SPTBN2: spectrin beta, non‐erythrocytic 2
UTR: untranslated region
VGKC: voltage‐gated potassium channel
VPS11: VPS11 core subunit of CORVET and HOPS complexes

## 1 INTRODUCTION

Hereditary ataxias are defined as a large group of diseases that have inherited cerebellar or spinocerebellar ataxia (SCA) and dysfunction as a core feature.^1^, ^2^ It is thus a clinical definition for a group of inherited neurodegenerative diseases, where (spino)cerebellar dysfunction can occur as an isolated sign or be a key feature of a more complex syndrome.

In a previous review article about hereditary ataxias in dogs,^1^ 5 categories were defined based on neuropathology: cerebellar cortical degenerations (CCDs), spinocerebellar degenerations, cerebellar ataxias without substantial neurodegeneration, canine multiple system degeneration (CMSD), and episodic ataxia. These diseases are described in several dog breeds, each with different ages of onset and speed of progression, combinations of different clinical signs and lesion topography in the central nervous system (CNS). As opposed to former reviews, the most recent human hereditary ataxia reviews now include some storage diseases if the key feature of the clinical presentation of the disease is (spino)cerebellar ataxia.^2^, ^3^ Cerebellar ataxia most commonly causes symmetric hypermetria (a form of dysmetria) that is characterized by sudden bursts of motor activity with a marked overflexion of the limbs on protraction without loss of strength.^4^ Spinocerebellar ataxia is described as more dancing or bouncing in quality.^5^ One of the major challenges of the current review was thus to define the boundaries of this large and heterogeneous group of diseases. Moreover, some diseases in dogs present differently from their human counterpart, fitting the definition of canine but not human hereditary ataxias, and vice versa.

This review summarizes the current knowledge about hereditary ataxias in dogs. As in previous reviews of hereditary ataxia in dogs,^1^, ^5^ diseases will still be grouped according to their neuropathological features. A new category, multifocal degenerations with predominant (spino)cerebellar component, was added in order to adapt for new hereditary ataxia syndromes that do not fit the definition of the previously existing categories, encompassing some specific neuroaxonal dystrophies (NADs) and lysosomal storage diseases which cause major (spino)cerebellar dysfunction. Those for which an association to a genetic variant has been identified are discussed in this article and summarized in Table 1, specific information about the variants is provided in File S1. For each of these diseases, an overview of the clinical presentation, diagnostic, histopathological findings, and genetic variant is given. The terminology (associated or causal) used in this review conforms to the terminology used in the original articles describing the variants. A standardized terminology as per the American College of Medical Genetics and Genomics (ACMG) guidelines^37^ is favored to describe variants when possible. Diseases and case reports without genetic information will not be discussed in this review but are listed in Table 2.

**TABLE 1:** Overview of hereditary ataxias in dogs with known genetic variant (all are homozygous recessive) or region of interest (adapted from Urkasemsin et al^1^).

Disease	Breed	Onset	Progression	Gene	Gene function	References
*Cerebellar cortical degenerations*						
Primary Purkinje cell degeneration	Beagle	3 weeks	Minimal	*SPTBN2*	CNS cytoskeleton integrity	Forman et al^6^
	Finnish Hound	4‐12 weeks	Rapid	*SEL1L*	Protein degradation and autophagy	Kyöstilä et al^7^
	Gordon Setter Old English Sheepdog	6‐10 months 6‐40 months	Slow	*RAB24*	Protein degradation and autophagy	Agler et al^8^
	Hungarian Vizsla	2‐3 months	Rapid	*SNX14*	Synaptic transmission and neuronal excitability	Fenn et al^9^
	Scottish Terrier	2 months‐6 years	Slow	*CFAX* ^a^	/	Urkasemsin et al^10^
Primary granule cell degeneration	Australian Working Kelpie	5‐12 weeks	Variable	*CFA3* ^a^ *CFA9* ^a^ *CFA20* ^a^	/	Shearman et al^11^ Wade et al^12^
*Spinocerebellar degenerations*						
Late‐onset ataxia SAMS	Jack and Parson Russel Terriers	6‐12 months 2‐6 months	Progressive Progressive	*CAPN1* *KCNJ10*	Protein degradation and autophagy Cation trafficking	Forman et al^13^ Gilliam et al^14^
SAMS	Smooth Haired and Toy Fox Terriers Patterdale Terrier	2‐6 months 3‐4 months	Progressive	*KCNJ10*	Cation trafficking	Rohdin et al^15^ Fisher and Liebel^16^
SAMS	Dachshund	8 weeks	Progressive	*KCNJ10*	Cation trafficking	Vanhaesebrouck et al^17^
SAMS/SDCA1 SAMS‐like	Belgian Malinois Shepherd	4‐8 weeks 3‐6 months	Progressive/rapid Slow	*KCNJ10* *SLC12A6*	Cation trafficking	van Poucke et al^18^ Mauri et al^19^ van Poucke et al^20^
SAMS	Bouviers des Ardennes	2‐6 months	Rapid/Progressive	*KCNJ10*	Cation trafficking	Stee et al^21^
Spinocerebellar ataxia 8	Alpine Dachsbracke	3 weeks	Rapid	*SCN8A*	Cation trafficking	Letko et al^22^
*Cerebellar ataxia without substantial neurodegeneration*						
	Coton de Tulear	2 weeks	Stable	*GRM1*	Cation trafficking	Zeng et al^23^
	Italian Spinone	4 months	Progressive	*ITPR1*	Cation trafficking	Forman et al^24^
	Norwegian Buhund	<12 weeks	Slow	*KCNIP4*	Cation trafficking	Jenkins et al^25^
*Multifocal degenerations with predominant (spino)cerebellar component*						
Canine multiple system degeneration	Kerry Blue Terrier	9 weeks to 5 months	Progressive	*SERAC1*	Mitochondrial function	Guo et al^26^
	Chinese crested	3‐6 months				
SDCA2	Belgian Malinois Shepherd	4 weeks	Rapid	*ATP1B2*	Cation trafficking	Mauri et al^27^
CNS atrophy and cerebellar ataxia		2 weeks	Rapid	*SELENOP*	Mineral transport	Christen et al^28^
Hereditary ataxia	Australian Shepherd	4‐19 months	Slow	*PLPLA8*	Mitochondrial function	Abitbol et al^29^
	Black Norwegian Elkhound	3‐4 weeks	Rapid	*HACE1*	Protein degradation	Bellamy et al^30^
Cerebellar degeneration and myositis complex	Nova Scotia Duck Tolling Retriever	10 weeks to 6 months		*SLC25A12*	Mitochondrial carrier	Christen et al^31^
Vacuolar neurodegeneration	Lagotto Romagnolo	4 months to 4 years	Progressive	*ATG4D*	Protein degradation and autophagy	Kyöstila et al^32^
Neuroaxonal dystrophy	Rottweiler	1‐2 years	Slow	*VPS11*	Protein degradation and autophagy	Lucot et al^33^
Neuronal ceroid lipofuscinosis 4A	American and Pit Bull Staffordshire Terriers	1.5‐9 years	Slow	*ARSG*	Mucopolysaccharide degradation	Abitbol et al^34^
Neuronal ceroid lipofuscinosis 10	American Bulldog	1‐3 years	Slow	*CTSD*	Protein degradation	Awano et al^35^
GM1 gangliosidosis	Portugese Water Dog	4‐6 months	Rapid	*GLB1*	Glycolipid degradation	Wang et al^36^

Abbreviations: SAMS, spinocerebellar ataxia with myokimia, seizures, or both; SDCA, spongy degeneration and cerebellar ataxia.

^a^ Region of interest.

**TABLE 2:** Overview of hereditary ataxias in dogs without known genetic variant (adapted from Urkasemsin et al^1^).

Disease name	Breed	Onset	Progression	Cases	References
*Cerebellar cortical degenerations*					
Primary Purkinje cell degeneration	Rhodesian Ridgeback^a^	2 weeks	Rapid	9 related cases	Chieffo et al^39^
	Labrador Retriever	9‐17 weeks 7 weeks 3.5‐6 months	Rapid Slow /	3 littermates 1 case 5 cases	Perille et al^38^ Bildfell et al^40^ Sen et al^41^
	Labrador Retriever	4 years	Slow	1 case	Bertalan et al^42^
	English Bulldog	10‐12 weeks	Progressive	3 related cases	Gandini et al^43^
	Portugese Podenco	2‐3 weeks	Rapid	2 littermates	van Tongeren et al^44^
	Azores Cattle Dog	<16 weeks	Progressive	2 littermates	Varejão et al^45^
	Miniature Schnauzer	3 months	Rapid	1 case	Berry and Blas‐Machado^46^
	Papillon	5 months	Slow	1 case	Nibe et al^47^
	Boxer	3 years and 3 months	Progressive	1 case	Gumber et al^48^
	Lagotto Romagnolo	5 weeks	Rapid and then stabilizes	1 case	Jokinen et al^49^
	Samoyed	4‐6 weeks	Rapid and then stabilizes	2 cases	de Lahunta et al^4^
	Bernese Mountain Dog^b^	4‐6 weeks	Progressive	7 related cases	Carmichael et al^50^
Primary granule cell degenerations	Rough Coated Collie	4‐8 weeks	Rapid	39 related cases autos recessive	Hartley et al^51^
	Jack Russel Terrier	2 weeks	Progressive	5 littermates	Coates et al^52^
	Border Collie	6‐8 weeks 4 months	Rapid Progressive	2 littermates 2 littermates	Gill and Hewland^53^ Sandy et al^54^
	Labrador Retriever	13 months	Progressive	3 littermates	Huska et al^55^
	Bavarian Mountain Dog	3 months	Slow	3 cases	Flegel et al^56^
	Coton de Tulear^c^	8 weeks	Rapid	3 cases	Tipold et al^57^
	Australian Kelpie	6 weeks	Slow	1 case	Huska et al^55^
	Italian Hound	3 months	Progressive	1 case	Cantile et al^58^
	Lagotto Romagnolo	13 weeks	Rapid	1 case	Jokinen et al^49^
	Ibizian Hound	4‐6 weeks	Progressive, may stabilize	3 cases autos recessive	de Lahunta et al^4^
*Spinocerebellar degenerations*					
Late onset spinocerebellar degeneration	Brittany Spaniel^d^	5‐11 years	Slow	8 cases 1 case	Higgins et al^59^ de Lahunta et al^4^
Central axonopathy	Scottish Terrier	10‐12 weeks	Progressive	3 related cases	van Ham et al^60^
Episodic ataxia	Bichon Frise	4 months	Progressive^e^	1 case	Hopkins and Clarke^61^
*Other neurodegenerative diseases causing cerebellar dysfunction as core feature*					
Neuroaxonal dystrophy	Collie sheep dog	2‐4 months	Progressive	6 cases	Clark et al^62^
Neuroaxonal dystrophy	Dachsie‐cross	Few weeks	Progressive	2 littermates	Pintus et al^63^
Neuroaxonal dystrophy	Boxer	1‐7 months	Progressive	/	Dewey et al^64^
Neuroaxonal dystrophy	German Shepherd	15 months	Progressive	/	Dewey et al^64^
Neuronal ceroid lipofuscinosis	Dachshund	3 years	Slow progressive	1 case	Cummings and de Lahunta^65^
GM2 Gangliosidosis	German Pointer	1 year	Progressive	1 case	Singer et al^66^

^a^ Associated to coat color dilution.

^b^ Nodular liver, abdominal varicosities, and ascites.

^c^ Presumed immune‐mediated.

^d^ Lesions in the medullary general proprioceptive nuclei and spinal cord dorsal horn neurons.

^e^ Complete resolution of signs was reported following treatment with 4‐aminopyridine.

## 2 CEREBELLAR CORTICAL DEGENERATIONS

CCD, or cerebellar abiotrophies, are 1 of the most common neurodegenerative diseases in animals. Two forms of CCD are described: primary Purkinje cell degeneration (most common) and primary granule cell or “granuloprival” degeneration. Classic signs include hypermetric and dysmetric ataxia, wide‐based stance, tendency to lean and fall towards both sides, difficulty negotiating stairs, loss of balance, intention tremors, and occasional nystagmus. Neurological examination can reveal postural reaction deficits, delayed hopping with an exaggerated response, bilateral absent menace responses, as well as positional spontaneous nystagmus and opsoclonus.^6^, ^7^, ^8^, ^12^, ^52^, ^58^, ^67^, ^68^, ^69^, ^70^, ^71^, ^72^

**FIGURE 1:**
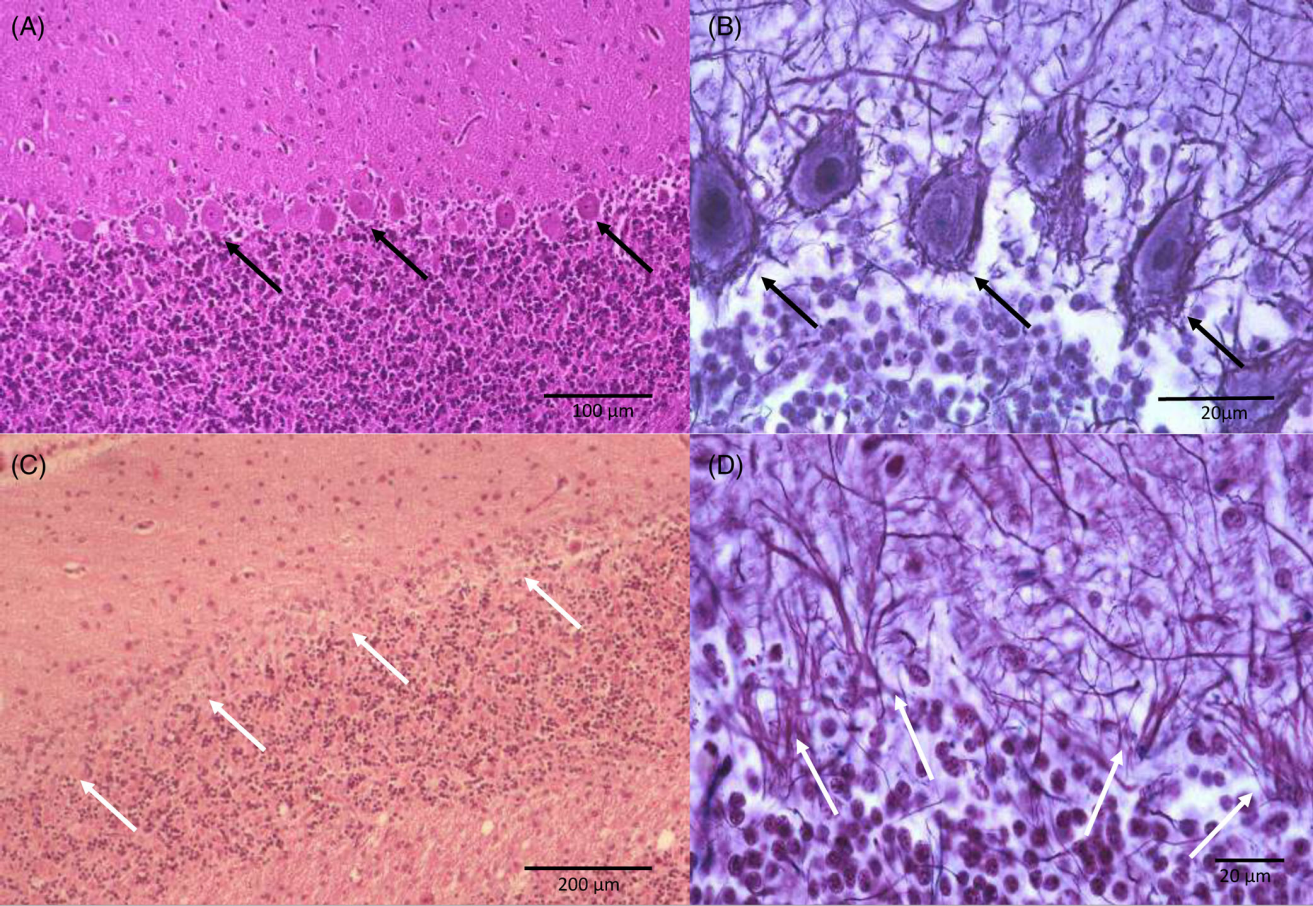
Histopathology of the cerebellum of a dog with primary Purkinje cell degeneration. (A,B) Cerebellum of a normal dog (for comparison) under hematoxylin eosin (HE) stain (A); and under silver impregnation (b). Purkinje cells are present and surrounded by basket cell processes (black arrows). (C,D) Cerebellum of a dog with primary Purkinje cell degeneration under HE stain (C) showing loss of Purkinje cells (white arrows), and under silver impregnation (D) with empty baskets (white arrows) and secondary shrinkage of the molecular layer. Courtesy of Martí Pumarola Battle.

**FIGURE 2:**
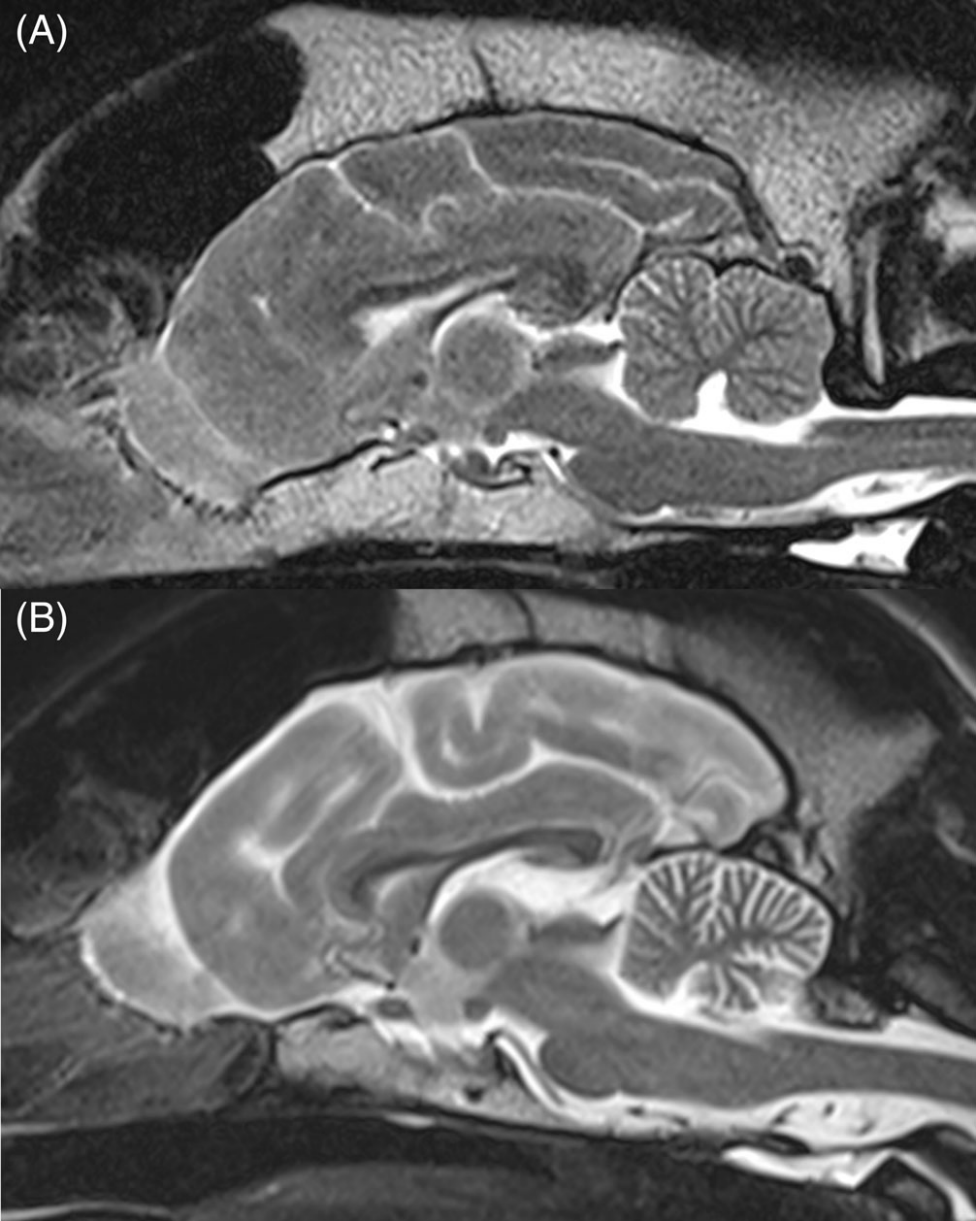
Sagittal T2W MRI image (1.5T) of the brain of a normal dog (A) and a Border Collie with cerebellar cortical degeneration showing marked diffuse atrophy of the cerebellar cortex, most obvious in the dorsal half of the cerebellum (B). Courtesy of Ariel Cohen Solal.

**FIGURE 3:**
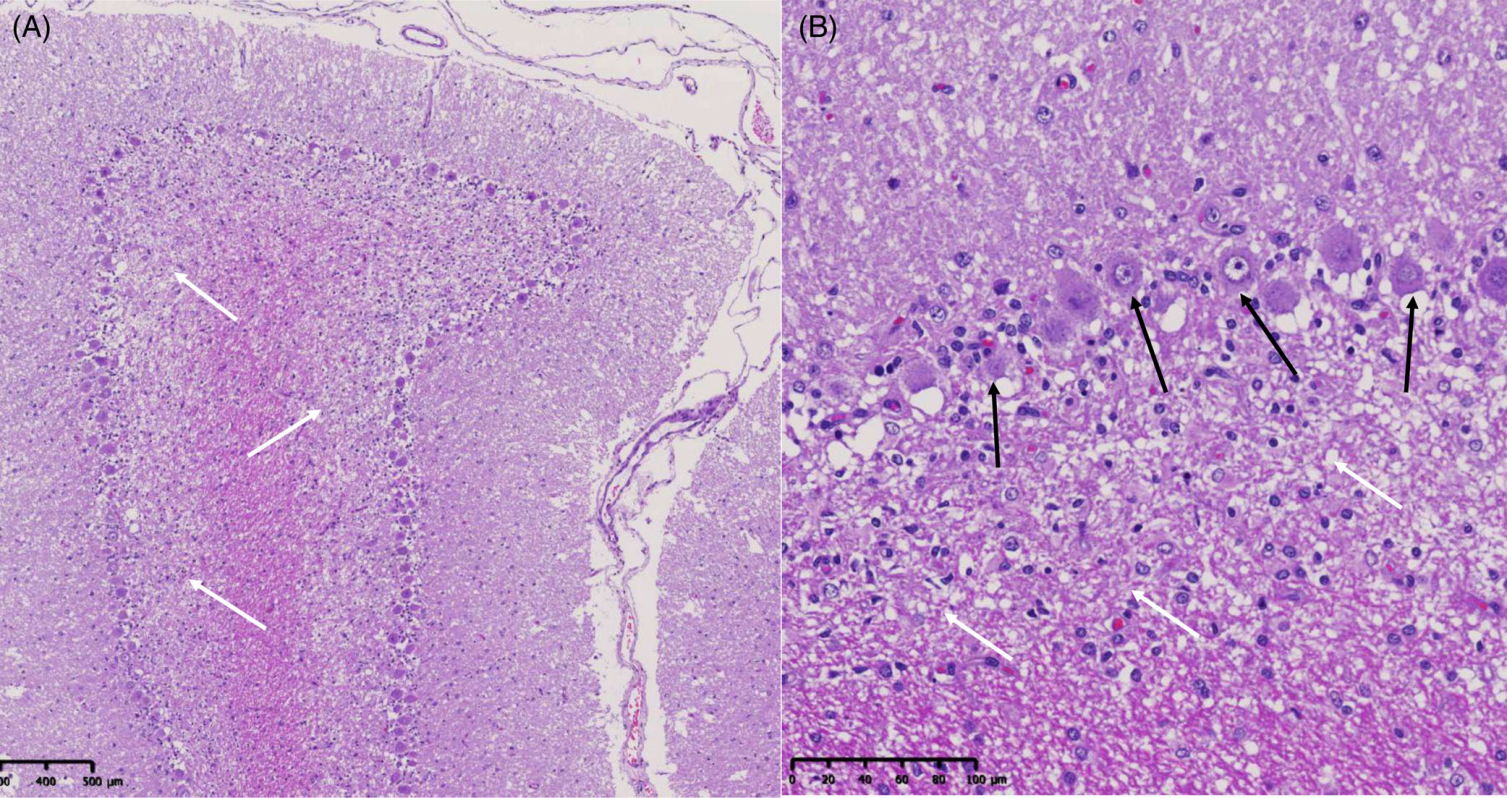
Histopathology of the cerebellum of a dog with granuloprival degeneration showing thinning of the granule cell layer because of a massive loss of granule cells (white arrows), with relative sparing of Purkinje cells (black arrows). Courtesy of Martí Pumarola Battle.

In primary Purkinje cell degeneration, the functional disturbance caused by the underlying genetic variant results in spontaneous premature death of Purkinje neurons (Figure 1). The clinical signs reflect the loss of the normal inhibitory function of the Purkinje neurons on cerebellar and brainstem nuclei. Signs can have a neonatal, juvenile, or sometimes adult onset, and can be progressive or static (after rapid cell destruction). On MRI, cerebellar atrophy is frequently seen in dogs with advanced clinical disease, being most obvious in the dorsal half of the cerebellum (Figure 2). As there is no relevant difference in the percentage of the brain occupied by the cerebellum among breeds in normal 1 to 5 years old dogs, the ratio between the brainstem and cerebellum midsagittal cross‐sectional area using a cut off value of 89% is a reliable tool to detect cerebellar atrophy.^73^ On necropsy, a macroscopically decreased size of the cerebellum is also seen in chronic cases, with a cerebellum to brain ratio of 6% to 9.7% (normal: 10%‐12%). Typical microscopic lesions for the primary Purkinje cell degeneration form of CCD include primary Purkinje cell loss, with secondary transneuronal retrograde degeneration of granule cells and shrinkage of the molecular layer. In the granuloprival degeneration form of CCD, the main lesions include a progressive loss of granule cells, thinning of the granule cell layer and gliosis, with relative sparing of Purkinje cells^6^, ^7^, ^8^, ^12^, ^52^, ^58^, ^67^, ^68^, ^69^, ^70^, ^71^, ^72^ (Figure 3). Genetic variants have so far only been associated with Purkinje cell degenerations, and have been described in the Beagle,^6^ Finnish Hound,^7^ Gordon Setter,^8^ Old English Sheepdog,^8^ and Viszla.^9^ Regions of interest have been identified in the Scottish Terrier^10^ (primary Purkinje cell degeneration) and Australian Kelpie^11^, ^12^ (granuloprival degeneration).

### 2.1 Beagle

Signs in Beagles are noticed at the onset of walking (3 weeks of age). Progression of signs is minimal, but the puppies are severely affected: falling frequently while trying to walk and having difficulties eating, frequently missing the food bowl when lowering their head to prehend the food.^69^, ^70^ An autosomal recessive 8‐bp deletion in *SPTBN2* (XM_038424853.1:c.5855_5862del (p.[Ile1952Argfs*28])) is associated with this disease, which compromises the functional expression of the spectrin beta, non‐erythrocytic 2 protein (SPTBN2), a cytoskeletal protein that is highly expressed in Purkinje neurons.^6^

### 2.2 Finnish Hound

The onset of signs in Finnish Hounds is described around 9 weeks (range: 4‐12 weeks) of age. Rapid progression of this disease results in euthanasia 4 weeks after onset of signs. An autosomal recessive missense variant in *SEL1L* (XM_038674398.1:c.1972T>C (p.[Ser658Pro])) is associated with this disease. Ten percent from the studied Finnish Hound population is carrier of this variant. This gene encodes the SEL1L adaptor subunit of ERAD E3 ubiquitin ligase, which is a component of the endoplasmic reticulum‐associated protein degradation.^7^

### 2.3 Gordon Setter and Old English Sheepdog

Clinical signs in Gordon Setters become apparent between 6 and 10 months of age, whereas a later onset of signs, between 6 and 40 months of age occurs in Old English Sheepdogs. The disease is slowly progressive (over several years) in both breeds, with milder severity in the Old English Sheepdog.^67^, ^68^, ^71^ In Gordon Setters, an increased extensor muscle tone is also reported with disease progression.^67^, ^68^ An autosomal recessive missense variant in *RAB24* (XM_038663268.1:c.113A>C (p.[Gln38Pro])) is associated with this disease in both breeds. This gene encodes RAB24, member RAS oncogene family, which is a GTPase.^8^

### 2.4 Hungarian Vizsla

Onset of signs in Vizslas occurs about 2 to 3 months of age, and rapid progression of signs results in euthanasia a few weeks later. An autosomal recessive splice donor variant in *SNX14* (XM_038684085.1:c.2713+1G>A), is associated with this disease. It compromises functional expression of the sorting nexin 14 (SNX14) protein.^9^

### 2.5 Scottish Terrier

Affected Scottish Terriers typically start showing signs between 2 months and 6 years of age (median: 7 months), with 76% of the dogs having an onset of signs before 1 year of age. The progression of the disease is very slow (over several years) in most dogs (74%) and signs can sometimes even stabilize after 1 year of age. The severity of the disease is variable in this breed, but very few affected dogs are euthanized because of their CCD.^74^, ^75^ On histopathology polyglucosan body accumulation is identified in the molecular layer of the cerebellum.^76^ The disease trait maps to a 4‐Mb region on chromosome X (CFAX) in this breed, but an association to a variant has not yet been identified.^10^

### 2.6 Australian Working Kelpie

Onset of clinical signs in Australian Working Kelpies usually occurs between 5 and 12 weeks of age. Clinical severity is quite variable in this breed, ranging from mild nonprogressive ataxia and intention tremors to severe ataxia and seizures.^11^, ^77^, ^78^ Histology in this breed reveals granuloprival degeneration, with secondary loss of Purkinje cells in severely affected individuals. The disease trait maps to a 3‐Mb region on chromosome 3 (CFA3), but an association to a variant could not be identified.^11^ More recently, another study found 2 more loci associated with CCD in this breed on CFA9 and CFA20, respectively. Interestingly, dogs homozygous for the risk haplotype on CFA20 show clinical signs before 10 weeks of age, whereas dogs homozygous for the risk haplotype on CFA9 have later onset ataxia.^12^

## 3 SPINOCEREBELLAR DEGENERATIONS

Spinocerebellar degenerations are, except for late‐onset ataxia (LOA), due to the dysfunction of an electrolyte channel. Clinically, SCA can present alone or as part of a neurological syndrome, and diagnostic tests typically do not reveal abnormalities. On histopathology, the most prominent lesions are found in the spinal cord, brainstem, and cerebellum, consisting of an axonopathy and spongy lesions (vacuoles) within the neuropil. This group encompasses LOA, SCA with myokymia, seizures, or both (SAMS) and spongy degeneration and cerebellar ataxia (SDCA) 1, SAMS‐like syndrome, and SCA8.

### 3.1 Late onset ataxia in the Jack and Parson Russell Terriers

This disease is most prominent in the Parson Russell Terrier breed but occurs in a few Jack Russell Terriers. Signs of dysfunction have an onset between 6 and 12 months of age, hence “late onset.” Clinically, dogs display progressive symmetric ataxia, truncal sway, pelvic limb hypermetria, and impaired balance. A certain degree of stabilization of the clinical signs with intermittent worsening can occur. In later stages, ambulation often becomes difficult, leading to euthanasia of most dogs. An autosomal recessive missense variant in *CAPN1* (XM_038425033.1:c.344G>A (p.[Cys115Tyr])) is associated with this disease. *CAPN1* encodes for the intracellular calcium‐dependent cysteine protease calpain 1 and is highly expressed in the brain and nervous system. This variant occurs in a small number of Jack Russell Terriers.^13^

### 3.2 Spinocerebellar ataxia with myokymia, seizures, or both (SAMS)

Hereditary ataxias have been recognized since the 1940s in the Smooth Haired Fox Terrier breed in Sweden^79^ and since the 1970s in the Jack Russell Terrier breed in the United Kingdom.^80^ Since, several reports have been published in the Jack and Parson Russell Terrier breeds,^14^, ^81^, ^82^, ^83^, ^84^, ^85^, ^86^ Smooth Haired and Toy Fox Terrier breeds,^15^, ^87^, ^88^ Malinois Shepherd breed,^18^, ^19^ as well as in the Patterdale Terriers^16^ and Bouvier des Ardennes breed.^21^ One affected Dachshund is reported.^17^

The SCA has a juvenile onset (2‐6 months of age in most breeds) and can occur alone or in combination with myokymia and neuromyotonia, seizures, or both. The SCA is usually mild at onset and progressively worsens to become severe by 6 months of age. It has a characteristic hypermetric and spastic nature (because of increased extensor tone) causing a typical dancing, bouncing gait with exaggerated abduction of the pelvic limbs^14^, ^15^, ^16^, ^18^, ^21^, ^79^, ^80^, ^81^, ^83^, ^84^, ^86^, ^88^ (Movie S1). Neurological examination is, besides for the ataxia, usually normal, although some cases have an absent menace response or pelvic limb proprioception deficits^16^, ^19^, ^83^, ^84^, ^88^ and intention tremors might become apparent in advanced stages.^16^, ^19^, ^80^, ^81^ Large dogs such as Malinois Shepherds and Bouviers des Ardennes are usually euthanized on welfare grounds between 5 and 11 months of age,^18^, ^19^, ^21^, ^89^ whereas up to 30% of small dogs such as Jack and Parson Russell Terriers and Smooth Haired Fox Terriers have been reported to still be alive by 4 years of age.^16^, ^86^, ^90^ The size and weight of the dog probably has an influence on its ability to cope with the severe ataxia, hence its ability to walk and the owner's decision to consider euthanasia.

**FIGURE 4:**
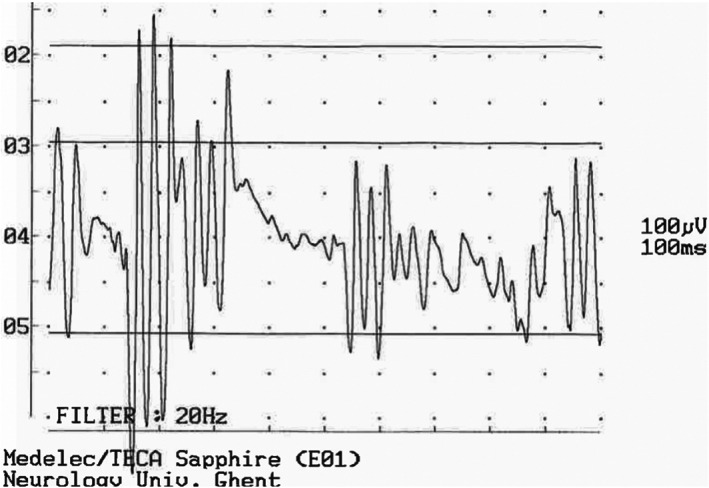
Electromyography of a Malinois dog with SAMS, showing neuromyotonic discharges in appendicular muscles clinically affected with myokymia.

Myokymia is seen in up to 70% to 75% of affected Jack and Parson Russell Terriers^84^, ^91^ and in both reported Patterdale Terriers cases,^16^ but is much less frequent in SHFTs, Malinois Shepherds and Bouviers des Ardennes with SAMS.^15^, ^18^, ^21^ It is characterized by rhythmic undulating muscle movements producing vermicular movement of the overlying skin (Movie S2). The myokymia episodes arise between 2 and 30 months of age. Frequency varies from a few episodes per week to a few episodes per month, but can occasionally occur on a daily basis. Episode duration varies from a few minutes to a few hours. Exercise, excitement, and hot weather are predisposing factors. Myokymia and neuromyotonia are often discussed together as most dogs suffering from myokymia develop neuromyotonic episodes (about 80% of dogs). The latter is characterized by muscle stiffness leading to a collapse into lateral recumbency without loss of consciousness, lasting from several minutes to several hours (Movie S3). Potentially life‐threatening hyperthermia (>43°C) and tachypnea are frequent during those neuromyotonic episodes, causing spontaneous death of some dogs. In about half of the dogs, myokymia, muscle stiffness, and collapse are preceded by intense facial rubbing. In a minority of dogs, neuromyotonia can precede myokymia.^84^ Electromyography of muscles clinically affected by myokymia shows myokymic or neuromyotonic discharges^18^, ^83^, ^84^, ^86^ (Figure 4). Epileptic seizures occur in 12.5% to 50% of dogs with SAMS.^14^, ^18^, ^83^, ^84^

**FIGURE 5:**
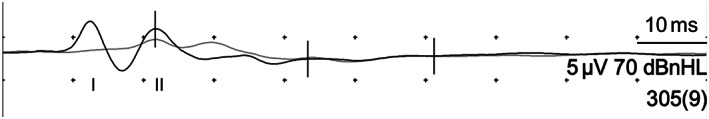
Brainstem auditory evoked recordings of a Bouvier des Ardennes dog with SAMS showing disappearance of waves III/VI and V.

**FIGURE 6:**
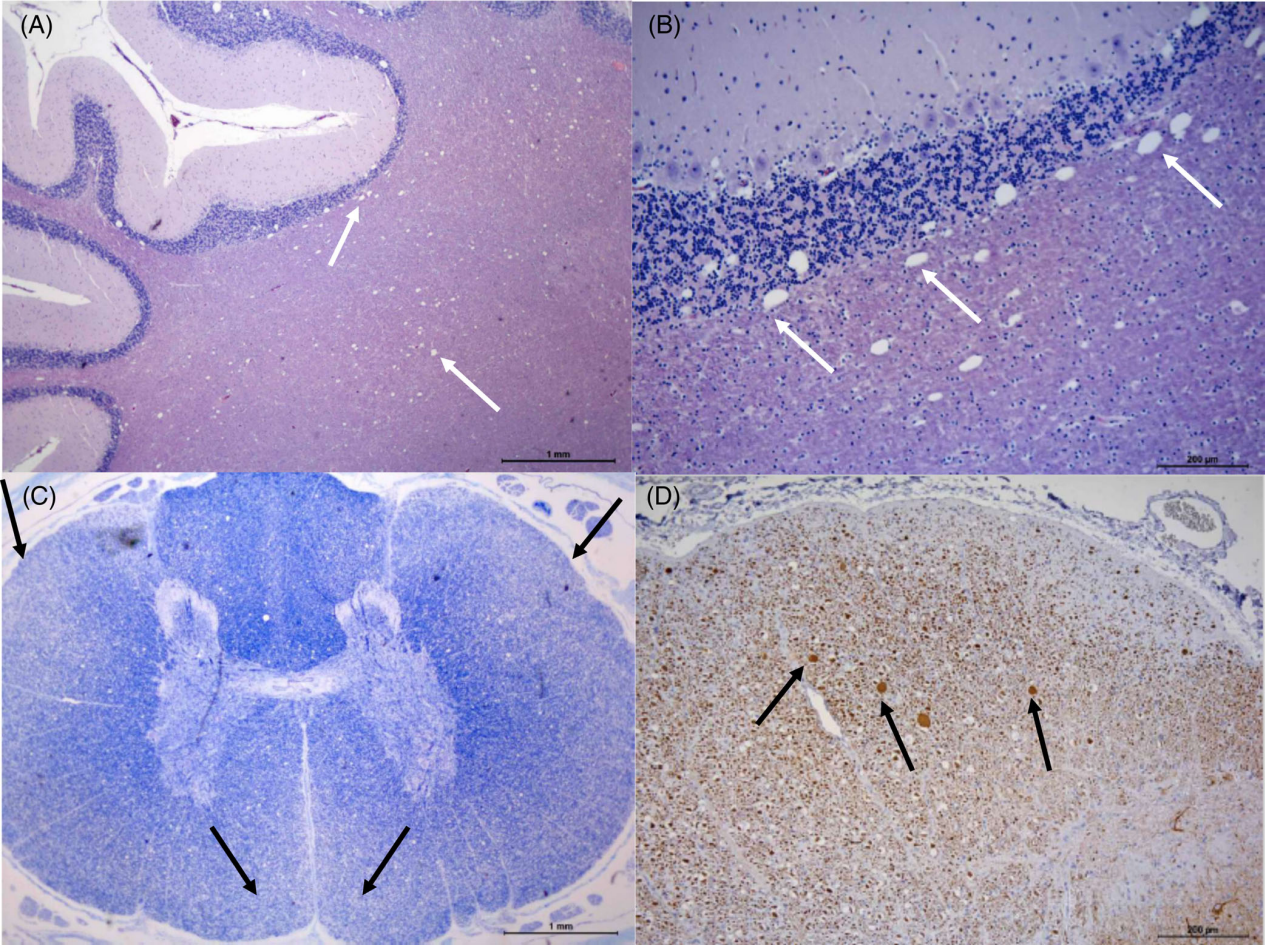
Histopathology of the spinal cord and cerebellum of a Bouvier des Ardennes dog affected with SAMS. (A,B) HE stain shows spongiosis (spongy degeneration) in the cortical white matter and infiltrating the cortical cerebellar granular layer (white arrows). (C) Luxol fast blue (LFB) stain of the spinal cord shows marked pallor of lateral and ventral funiculi (black arrows). (D) IHC of dorsal spinocerebellar tract of cervical spinal cord showing decreased immune positivity for NF200, loss of axonal component and presence of some spheroids (black arrows). Courtesy of Martí Pumarola Battle.

Brainstem auditory evoked recordings of these dogs show loss of waves III, IV, and V, as well as in most cases mildly increased latencies for waves I and II^17^, ^18^, ^82^, ^83^ (Figure 5). Histopathology reveals a bilateral symmetrical myelopathy and severe degenerative changes in the CNS auditory pathways (Figure 6). The myelopathy is characterized by a predominant axonopathy with myelin swelling, vacuolization, and astrogliosis (most severe in the dorsolateral and ventromedial funiculi of the cervical spinal cord). A common feature is loss of myelinated axons and diffuse gliosis in the central auditory pathways most severely affecting the dorsal nucleus of the trapezoid body, but also involving the trapezoid body, cochlear nuclei, and lateral lemniscus. These degenerative CNS auditory pathway changes are not described in Smooth Haired Fox Terrier, where the lesions appear limited to the spinal cord.^14^, ^15^, ^18^, ^19^, ^79^, ^80^, ^87^, ^88^

SAMS is typically associated with variants in *KCNJ10*, which encodes for the potassium inwardly rectifying channel subfamily J member 10 (KCNJ10), a voltage‐gated potassium channel (VGKC) that is mainly expressed in the brain, spinal cord, inner ear, and kidneys. Several breed‐specific variants have been described in dogs. Phenotypic heterogeneity is well documented with *KCNJ10* variants in dogs.^15^, ^18^, ^19^, ^21^, ^84^ In dogs, variants in *KCNJ10* seem to be highly associated with SCA and not with myokymia and neuromyotonia, as all dogs homozygous for a *KCNJ10* variant have SCA but not all of them have myokymia and neuromyotonia. Moreover, no *KCNJ10* variant has yet been found in dogs with myokymia and neuromyotonia without SCA, suggesting that another variant (that has not been described yet) is probably associated with myokymia and neuromyotonia.^91^

#### 3.2.1 Jack and Parson Russell Terriers, Smooth‐Haired and Toy Fox Terriers, Patterdale Terriers

In the Smooth Haired Fox Terrier, a truncal sway, behavioral changes (anxiety, increased vocalization), and nose rubbing can occur in addition to the typical clinical signs. In this breed, progression is rapid at first, then slows down and alternates with periods of stabilization of signs, until the dogs become nonambulatory. Seizures have not been reported in Smooth Haired Fox Terriers, but some dogs have teeth chattering which could represent a form of focal seizure activity.^15^, ^79^, ^87^, ^88^

In Jack and Parson Russell Terriers, Smooth Haired and Toy Fox Terriers as well as Patterdale Terriers, an autosomal recessive *KCNJ10* missense variant (XM_038448705.1:c.627C>G (p.[Ile209Met])) is the main genetic variant associated with SAMS.^14^, ^15^, ^16^, ^84^, ^91^ This is likely because Jack and Parson Russell Terriers are closely related British hunting breeds share common ancestry with the Smooth Haired Fox Terrier. However, some affected Jack and Parson Russell Terriers from 1 study^90^ as well as 2 Jack Russell Terriers, from another study,^84^ did not carry this *KCNJ10* variant (nor the *CAPN1* variant), suggesting that at least 1 additional variant is segregating in the Jack and Parson Russell Terrier breeds. An additional *KCNJ10* variant in the 3′‐UTR region (a C‐insertion with a predicted regulatory effect on the expression) was described in some of these unexplained cases,^90^ but this variant was also homozygously present in a clinically healthy control dog of more than 4 years old.

#### 3.2.2 Belgian Malinois Shepherd and Bouvier des Ardennes

In both breeds, spinocerebellar degeneration can present with a spectrum of phenotypic severity, ranging from the classical SAMS phenotype^18^, ^21^ to a severe cerebellar phenotype reported as SDCA1.^19^, ^21^, ^89^, ^92^ In both phenotypes, the wide‐based stance and generalized SCA typically develops around 4 to 8 weeks of age. In the SAMS phenotype, the SCA is milder at first and progresses more slowly, resulting in a nonambulatory status by 5 to 6 months of age in Malinois Shepherds and 7 to 11 months of age in Bouvier des Ardennes dogs (Movie S4). Myokymia, neuromyotonia, and seizures can also be part of this picture, and the main abnormal findings on neurological examination are absent menace responses and patellar reflexes.^18^, ^21^ In SDCA1, the severity of the SCA is more pronounced at onset and progresses faster (Movie S5). Severe intention tremors, truncal sway, stumbling, staggering, bunny hopping, loss of balance, and falling are also seen with affected dogs becoming nonambulatory by 2 to 4 months of age, precipitating euthanasia. Myokymia, neuromyotonia, and seizures have not been reported in SDCA1, and neurological examination can also show delayed proprioception. On histopathology, main lesions include spongy degeneration of the cerebellar nuclei, granular cell layer, cerebellar white matter, vestibulocochlear tract, and reticular formation, as well as a bilateral symmetrical demyelinating myelopathy targeting the dorsolateral spinocerebellar tracts.^18^, ^19^, ^21^, ^89^ SAMS and SDCA1 are associated with the exact same autosomal recessive *KCNJ10* missense variant (XM_038448705.1:c.986T>C (p.[Leu329Pro])) in both Malinois Shepherd and Bouvier des Ardennes dogs. The fact that the same variant was found in both breeds is likely explained by the fact that some Malinois Shepherd dogs were used to expand the Bouvier des Ardennes bloodlines after this breed nearly became extinct at the beginning of the 20th century.^21^

### 3.3 SAMS‐like syndrome in the Belgian Malinois Shepherd

**FIGURE 7:**
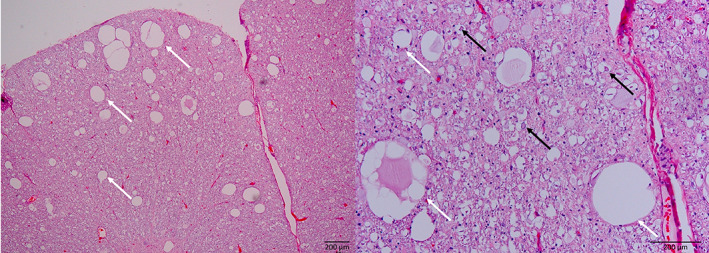
Histopathology of the spinal cord of a Malinois dog affected with SAMS‐like syndrome. H&E of the cervical spinal cord showing vacuolation (white arrows) and axonopathy of the ventral pathways. The vacuoles are optically empty or contain an eosinophilic fluid (consistent with axoplasm) and several axonal spheroids (black arrows) are present. Courtesy of Leslie Bosseler.

Ataxia in this disease has an onset between 3 and 6 months of age. Clinical signs include a severe generalized spinocerebellar ataxia (worse on the pelvic limbs), mild palmigrade stance, and moderate paraparesis (Movie S6). Patellar reflexes are typically absent, and myokymia‐like muscle contractions can be seen in the sedated patient. The disease has a slow progressive course, resulting in a nonambulatory status (and subsequent euthanasia) by 2.5 to 3 years of age. Neuropathological lesions include bilateral symmetrical axonopathy of the white matter affecting the whole spinal cord but most severe in the ventral corticospinal, vestibulospinal, lateral corticospinal, and dorsal and ventral spinocerebellar tracts (Figure 7). The ventral nerve roots, brainstem, and cerebellum can also be affected. An autosomal recessive deletion‐insertion sequence change (delins) in *SLC12A6* (XM_038441820.1:c.178_181delinsCATCTCACTCAT (p.[Met60Hisfs*14])) is associated with this disease. It compromises the functional expression of the solute carrier family 12 member 6 protein (SLC12A6), a K^+^‐Cl^−^ co‐transporter that is highly expressed in the brain, spinal cord and peripheral nerves. This variant was only found in a single family of Malinois dogs, therefore it likely is a private variant because of founder effect.^20^

### 3.4 SCA8 in the Alpine Dachsbracke

This disease has an onset of signs when puppies start to move, around 3 weeks of age. Clinical signs include severe cerebellar ataxia, intention tremors, loss of balance, and falling, resulting in euthanasia by 10 to 12 weeks of age. Histopathologic lesions include spongy degeneration throughout the white and gray matter of the brain and optic chiasm, as well as marked astrogliosis and axonal degeneration most severely affecting the vestibulocochlear nucleus, cerebellar nuclei, thalamus, and brainstem. An autosomal recessive *SCN8A* missense variant (XM_038438133.1:c.4898G>T (p.[Gly1633Val])) is associated with this disease. *SCN8A* encodes the sodium voltage‐gated channel alpha subunit 8 (SCN8A), part of a voltage‐gated sodium channel that is expressed in both the CNS and peripheral nervous system (PNS).^22^

## 4 CEREBELLAR ATAXIAS WITHOUT SUBSTANTIAL NEURODEGENERATION

This group of diseases demonstrates prominent clinical cerebellar dysfunction in the absence of degenerative changes on histopathology. Disease is the consequence of a functional issue in the cerebellum. Genetic variants responsible for these diseases are so far all located in genes encoding proteins involved in cation trafficking.

### 4.1 Neonatal cerebellar ataxia in the Coton de Tulear

Severe clinical signs are visible from 2 weeks of age and are nonprogressive. These include head titubation, intention tremors, severe ataxia resulting in inability to stand and the use of propulsive movements (“swimming”) to move around. Pups frequently fall into lateral recumbency with paddling and intermittent decerebellate posture. Neurological examination reveals fine vertical eye tremors, positional nystagmus, absent menace response, and severely decreased proprioception. There are no microscopic lesions but evidence of synaptic dysfunction in the cerebellar molecular layer is found at an ultrastructural level.^93^ An autosomal recessive truncated retrotransposon insertion in *GRM1* (XM_038654394.1:c.2331_2332insN[62]) is associated with this disease, as it compromises the functional expression of the glutamate metabotropic receptor 1.^23^

### 4.2 Spinocerebellar ataxia in the Italian Spinone

Clinical signs in Italian Spinone dogs have an onset about 4 months of age and include a wide‐based stance, hypermetric gait, truncal sway, impaired balance, pendular nystagmus, absent menace response as well as intention tremors in advanced cases. Progression of the vestibular signs result in a nonambulatory status and euthanasia at 1 year. No degenerative changes are present on histopathology, but Purkinje cells show a markedly lower expression of calbindin D‐28K and inositol triphosphate receptor 1 (ITPR1) than in normal dogs. An autosomal recessive intronic GAA repeat expansion in *ITPR1* (NC_051824.1:g.12923647CTT[7_651]), which compromises functional protein expression, is associated with this disease.^24^

### 4.3 Hereditary ataxia in the Norwegian Buhund

Onset of signs in Norwegian Buhunds occurs before 12 weeks of age. Clinical signs are slowly progressive and include a broad‐based stance, cerebellar ataxia, and fine head tremors. Neurological examination reveals a reduced menace response. On histopathology, Purkinje cell loss is very mild with marked decreased expression of calbindin D‐28K and ITPR1, and a reduction of the expression of (VGKC) interacting protein 4 (KCNIP4) throughout the entire cerebellar cortex.^94^ An autosomal recessive *KCNIP4* missense variant (XM_038662081.1: c.487T>C (p.[Trp163Arg])) is associated with this disease. *KCNIP4* encodes the potassium voltage‐gated channel interacting protein 4 (KCNIP4).^25^

## 5 MULTIFOCAL DEGENERATIONS WITH PREDOMINANT (SPINO)CEREBELLAR COMPONENT

This new category is defined as cerebellar or spinocerebellar degeneration with additional involvement of other parts of the CNS or other systems. These dogs clinically present with cerebellar or spinocerebellar dysfunction as a core feature, even though several of these diseases have additional clinical features because of their multifocal involvement. It encompasses CMSD, spinocerebellar degeneration and ataxia 2 (SDCA2), and CNS atrophy with cerebellar ataxia in Belgian Malinois Shepherds, Hereditary ataxia in Australian Shepherds and Black Norwegian Elkhounds, cerebellar degeneration‐myositis complex (CDMC) in Nova Scotia Duck Tolling Retrievers, vacuolar neurodegeneration in the Lagotto Romagnolo, NAD in the Rottweiler, neuronal ceroid lipofuscinosis (NCL) 4A in the Pitbull and American Staffordshire Terrier, and NCL10 in the American Bulldog, as well as GM1‐gangliosidosis in the Portuguese Water Dog.

### 5.1 Canine multiple system degeneration in the Kerry Blue Terrier and Chinese Crested dog

This disease is also called striatonigral and cerebello‐olivary degeneration because of the extrapyramidal nuclei (olivary nuclei, substantia nigra, putamen, and caudate nuclei) degeneration next to the CCD. This disease has an onset of signs between 9 weeks and 6 months of age. Dogs initially present a mild intention tremor and stiffness in thoracic limb gait, which progresses within 3 to 4 months to severe hypermetric ataxia, spasticity, truncal sway, wide‐based stance, delayed postural reactions, and decreased menace response. Signs progress to akinesia, inability to stand and euthanasia by 1 to 2 years of age.^4^, ^95^, ^96^, ^97^, ^98^ Cerebellar atrophy and T2W hyperintensity at the level of the caudate nuclei, putamen, and substantia nigra are visible on MRI in dogs affected for several weeks.^4^, ^98^

Macroscopic changes in advanced cases include a decreased cerebellar size (6%‐9% of total brain weight) and necrosis of the caudate nuclei, putamen, and substantia nigra. Microscopically, an ischemic degeneration of Purkinje cells is seen first, followed by Purkinje cell and secondary granule cell loss. With chronicity, degeneration occurs in the olivary nuclei, followed by acute bilateral degeneration of caudate nuclei and substantia nigra neurons. It is hypothesized that degeneration of the Purkinje neurons and caudate nuclei could be related to a toxic excessive glutamate accumulation. Degeneration of granule cells and olivary nuclei are retrograde to Purkinje cell degeneration, and degeneration of the substantia nigra is retrograde to caudate nuclei degeneration.^4^, ^95^, ^96^, ^97^, ^98^ This disease has been associated with 2 distinct breed‐specific autosomal recessive variants in *SERAC1*, respectively a nonsense variant (XM_038654522.1:c.1536G>A (p.[Trp512*])) in the Kerry Blue Terrier and a 4‐bp deletion (XM_038654522.1:c.182+1_182+4del) in the Chinese Crested,^26^ both compromising functional expression of the serine active site containing 1 protein (SERAC1).

### 5.2 SDCA2 in the Belgian Malinois Shepherd

Onset of signs in the Belgian Malinois Shepherd characterized by severe generalized spinocerebellar ataxia occurs at 4 weeks of age. Quickly, dogs also develop seizures, pacing, circling, and central blindness. The progression is rapid, causing all dogs to become nonambulatory by 6 weeks of age, resulting in euthanasia. On histopathology, lesions include bilateral symmetrical vacuolation of the neuropil at the level of the cerebellar nuclei, ventral horn gray matter of the spinal cord (most severe at the cervical intumescence) and brainstem. Neuronal necrosis and severe gliosis are also seen along the vacuolation in the spinal cord, as well as in the hippocampus, caudate nucleus, and cortex. An autosomal recessive *ATP1B2* SINE insertion variant (XM_038665439.1:c.130_131ins[LT796559.1:g.50‐276]) is associated with this disease. This gene encodes the ATPase Na^+^/K^+^ transporting subunit beta 2 protein (ATP1B2), an Na^+^/K^+^ ATPase from which the main role of which is to restore extracellular K^+^ homeostasis after neuronal depolarization and is predominantly expressed in the cerebellum.^27^

### 5.3 CNS atrophy with cerebellar ataxia in the Belgian Malinois Shepherd

Clinical signs have an onset around 2 weeks of age and are rapidly progressive but of variable severity. They include intention tremors, truncal wobbling, increased muscle tone with short episodes of spastic fits, reduced swallow reflex, difficulties walking as well as a lower body weight gain compared with healthy littermates. Euthanasia is performed in most dogs around 4 weeks of age because of severe ataxia, even though 1 dog was able to reach 10 years of age. Total selenium concentration in the blood of affected puppies is 30% lower than in healthy littermates. On histopathology, all layers of the cerebellum are atrophic with depletion of Purkinje cells and granule cells. Neuroaxonal degeneration is also present in the midbrain, brainstem, and spinal cord. Myelin content is severely diminished in the white matter of the brain and spinal cord. An autosomal recessive deletion of the complete *SELENOP* gene (NC_051808.1: g.67456991_67473571del) is associated with this disease. This gene encodes the selenoprotein P (SELENOP), a protein required for the transport of selenium into the CNS. It is hypothesized that the amount of dietary selenium intake might have an influence on the clinical variability.^28^

### 5.4 Hereditary ataxia in the Australian Shepherd

Clinical signs in Australian Shepherds are first noticed between 4 and 19 months of age and include moderate hypermetric ataxia that is worse on the pelvic limbs, bunny hopping, difficulties to stand up as well as negotiating stairs. Some dogs also present discrete intention tremors and absent patellar reflexes. A slow progression causes these dogs to become unable to walk around 30 to 44 months of age (Movie S7). Neurological examination at that stage reveals nonambulatory tetraparesis up to tetraplegia, severe spasticity of the hind limbs, proprioceptive deficits on all 4 limbs, and absent menace response in some dogs. MRI of the brain and cervical spinal cord is normal in early stages, but cerebellar atrophy is visible in advanced stages of the disease (by 72 months of age). Histopathology reveals diffuse brain demyelination and oligodendrogliosis. An autosomal recessive *PNPLA8* (XM_038423736.1:c.1169_1170dup (p.[His391Phefs*4])) frameshift variant was found to be associated with this disease.^29^ The patatin‐like phospholipase domain containing 8 protein (PNPLA8) contributes to mitochondrial function through its role in mitochondrial energy production.^99^ The allele frequency in Australian Shepherds was found to be 4.7% in the studied sample from the population in France.^29^

### 5.5 Hereditary ataxia in Black Norwegian Elkhound

This disease has an onset of signs around 3 to 4 weeks of age and is rapidly progressing. Clinical signs consist of a kyphotic posture, broad‐based stance, hypermetric ataxia with weakness worse on the pelvic limbs and a hanging tail (atypical for the breed). Neurological examination reveals abnormal postural reactions. Histopathology reveals axonal swellings in the cerebellar granule cell layer, spheroids in the Purkinje cell axons, and vacuoles in the brainstem white matter and nuclei. An autosomal recessive *HACE1* (XM_038684251.1:c.1001del (p.[Gly334Valfs*34])) single base pair deletion is associated with this disease. HACE1 (HECT domain‐ and ankyrin repeat‐containing e3 ubiquitin protein ligase 1) has an important role in the degradation of small proteins.^30^

### 5.6 CDMC in the Nova Scotia Duck Tolling Retriever

This disease has an onset of signs between 10 weeks and 6 months of age. It consists of generalized hypermetric ataxia that is more pronounced in the pelvic limbs and neuromuscular weakness, and can sometimes be associated with intention tremors. Some dogs show more pronounced muscular weakness, whereas others show more pronounced cerebellar signs. Neurological examination can reveal absent menace responses and reduced withdrawal reflexes in all 4 limbs. Blood examination shows a high serum creatine kinase (CK; >3× upper limit). MRI of the brain shows bilateral symmetrical lesions in the cerebellum and multifocal lesions in the masticatory muscles. Muscle biopsies reveal a fiber‐invasive lymphohistiocytic myositis without evidence of intracellular infectious agents. Histopathological findings include severe cerebellar nuclear degeneration. An autosomal recessive missense variant in the *SLC25A12* gene (XM_038447060.1:c.1370C>T (p.[Pro457Leu])) is associated with this disease. A carrier frequency of 7.1% and 2.7% is described in the European and North American Nova Scotia Duck Tolling Retriever studied population sample, respectively. *SLC25A12* encodes the solute carrier family 25 member 12 (SLC25A12), a mitochondrial aspartate/glutamate carrier.^31^

### 5.7 Vacuolar neurodegeneration in the Lagotto Romagnolo

This disease in Lagotto Romagnolos has an onset of signs between 4 months and 4 years of age (mean: 23 months). Clinical signs include clumsiness, cerebellar ataxia, episodic nystagmus (often the first sign to manifest), and behavioral changes. Delayed hopping and reduced or absent patellar reflexes are typical findings on neurological examination, whereas a reduced menace response and positional nystagmus are found in some cases. Disease progression is variable, leading to euthanasia months to years after onset of signs. On MRI, a mild atrophy of the cerebellum and forebrain and a small corpus callosum can be seen. Typically, histopathology reveals widespread profound neuronal cytoplasmic vacuolization in both the CNS and PNS, most severe in the deep cerebellar and brainstem nuclei. Purkinje and granule cell loss, as well as spheroid formation in the cerebellar white matter, thalamic, brainstem and cerebellar nuclei, and dorsal funiculus of the spinal cord are also seen. Cytoplasmic aggregation of vacuoles also occurs in secretory epithelial tissues (salivary glands and pancreas) and mesenchymal cells. An autosomal recessive missense variant *ATG4D* (XM_038428641.1:c.1372G>A (p.[Ala458Thr])) is associated with this disease, it encodes the autophagy related 4D cysteine peptidase (ATG4D).^32^, ^100^

### 5.8 Neuroaxonal dystrophy in the Rottweiler

**FIGURE 8:**
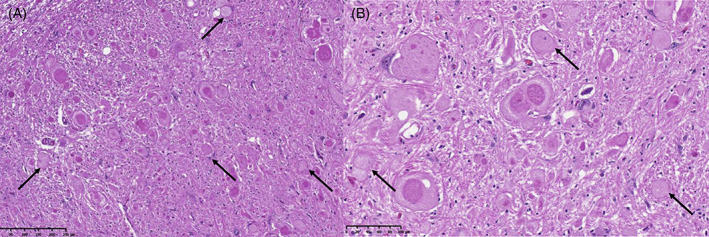
Histopathology of the gracilis nucleus of a Rottweiler dog with neuroaxonal dystrophy, showing the widespread presence of large degenerated neuronal bodies and swollen axons (spheroids, white arrows). Courtesy of Martí Pumarola Battle.

Affected Rottweilers usually start showing signs between 1 and 2 years of age and have a slow progressive (months to years) disease course. Thoracic limb hypermetria is the first sign, progressing into generalized hypermetric ataxia (worse on thoracic limbs), reluctance to jump, difficulty maintaining balance, head incoordination, and tremors. An absent menace response is a consistent feature. Spontaneous and positional nystagmus is seen in advanced cases. On histopathology, typical lesions of NAD include the widespread presence of swollen axons (spheroids) in the dorsal horns of the spinal cord, gracilis, and cuneatus nuclei, vestibular, lateral, and medial geniculate nuclei (Figure 8) and a decreased number of Purkinje cells.^72^, ^101^, ^102^, ^103^, ^104^ The accumulation of several synaptic proteins in dystrophic axons has been shown.^104^ An autosomal recessive *VPS11* missense variant (XM_038664676.1:c.2504A>G (p.[His835Arg])) is associated with this disease. It encodes the VPS11 core subunit of CORVET and HOPS complexes (VPS11), which is thought to affect the autophagic and other lysosomal pathways. The allele frequency in Rottweilers has been estimated at 3% in the European population.^33^

### 5.9 Lysosomal storage diseases targeting the cerebellum

#### 5.9.1 NCL4A in the American Staffordshire and Pit Bull Terriers

**FIGURE 9:**
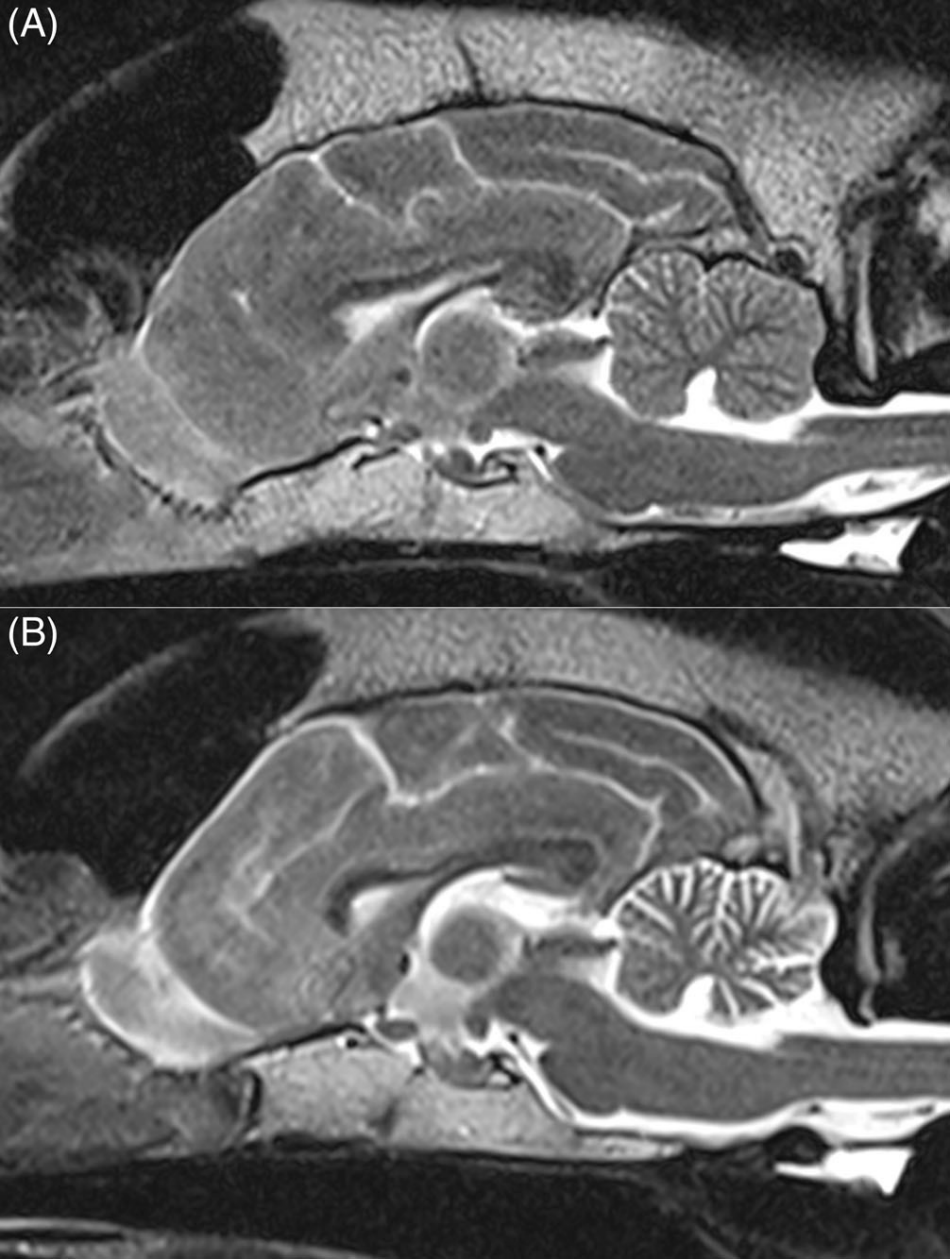
Sagittal T2W MRI image (1.5T) of the brain of a normal dog (A) and of an American Staffordshire Terrier with neuronal ceroid lipofuscinosis 4A, showing marked diffuse atrophy of the cerebellar cortex (B). Courtesy of Ariel Cohen Solal.

Signs of neurological dysfunction usually become evident between 3 and 6 years (range: 18 months to 9 years) of age in American Staffordshire and Pit Bull Terriers. Affected offspring from at least 1 affected parent have an onset of signs that occurs 1.5 years earlier than in the affected parent. The disease has a slow progression (in bursts, with periods of stabilization in between) and typical signs include stumbling, truncal sway, and ataxia (worse on pelvic limbs), difficulty negotiating stairs, progressing to dysmetric ataxia, nystagmus, coarse intention tremor, variable loss of menace response, marked truncal sway and falling with transient opisthotonus (Movie S8). The progression of clinical signs to the point where affected dogs became nonambulatory occurred in most dogs within 2 to 4 years after onset of signs (range: 6 months to 6.5 years). MRI demonstrates diffuse cerebellar atrophy (Figure 9) and cerebrospinal fluid can show a mild increase in protein.^105^ Brainstem auditory evoked recordings can show decreased amplitudes of waves I and II and increased inter‐wave latency for waves III to V.^106^ On histopathology, obvious cerebellar atrophy is present macroscopically (cerebellum to brain ratio: 5%‐7% [normal 10%‐12%]).^108^ Marked loss of Purkinje neurons, with secondary thinning of the molecular and granular layers as well as increased cellularity in the cerebellar nuclei, are visible under the microscope. Neuronal and vacuolar degeneration are present respectively in several thalamic and vestibular nuclei. Fluorescent lipopigment is present in both degenerating Purkinje cells and thalamic neurons.^108^ This disease was initially described as a CCD,^105^, ^106^, ^107^, ^109^ but is currently recognized as a NCL,^34^, ^108^, ^110^ even though some authors believe it should be reclassified as a mucopolysaccharidosis.^111^

An autosomal recessive missense variant in *ARSG* (XM_038674991.1:c.296G>A (p.[Arg99His])) is associated with this disease. This variant causes a 75% decrease of function of arylsulfatase G,^34^ which has a key role in the catabolism of mucopolysaccharide heparan sulfate.^111^ Penetrance of this variant is variable as in rare cases (1%‐3% of dogs) it can cause subclinical disease (no clinical signs but presence of mild accumulation of substrate in Purkinje neurons). As many as 50% healthy carriers were found in the American Staffordshire Terrier population.^34^

#### 5.9.2 NCL10 in the American Bulldog

Onset of clinical signs in American Bulldogs usually shows around 1.5 years of age but can occur between 1 and 3 years of age. The disease course is slowly progressive (several years), leading to euthanasia by about 5 years of age (range: 3.5‐5.5 years). Clinical signs include a wide based stance, hypermetric ataxia in all 4 (worse on pelvic) limbs and paraparesis, progressing to nonambulatory tetraparesis. Decreased proprioception, initially on the pelvic limbs and progressing to absent proprioception in all 4 limbs can be found on neurological examination. On histology, accumulation of autofluorescent cytoplasmic inclusions within neurons is seen throughout the entire CNS and the retinae, but is most severe in brainstem proprioceptive nuclei and spinal cord gray matter.^112^ An autosomal recessive missense variant in *CTSD* (NM_001025621.1:c.597G>A (p.[Met199Ile])) is associated with this disease. It induces a reduction of 36% of the normal enzymatic activity of the lysosomal proteinase cathepsin D in the brain of affected dogs. The allelic frequency in the studied American Bulldog population is 28%.^35^

#### 5.9.3 GM1‐gangliosidosis in the Portuguese Water Dog

Onset of signs in this breed occurs between 4 and 6 months of age. Clinical signs are rapidly progressive (over several weeks) and consist of wide‐based stance, dysmetric ataxia (worse on pelvic limbs), difficulty negotiating stairs, mild limb weakness, intention tremors, nystagmus, and possible lameness. Absent menace responses and delayed proprioception can be found on examination. Affected dogs are usually euthanized on welfare grounds by 6 to 7 months of age. On histopathology, a pale granular cytoplasm (GM1‐ganglioside) is present in neurons throughout the cerebral cortex, cerebellum, basal nuclei, brainstem, and spinal cord.^113^ Numerous cytoplasmic vacuoles can also be found in selected cells of various organs as the eyes, lymph nodes, thymus, lungs, liver, pancreas, spleen, kidneys, adrenal glands, and ovaries.^114^ An autosomal recessive missense variant in *GLB1* (NM_001037641.1:c.179G>A (p.[Arg60His])) is associated with GM1‐gangliosidosis in this breed.^36^ *GLB1* encodes the galactosidase beta 1, a lysosomal hydrolase that is found in the brain, plasma, and leucocytes. Pathogenic variants are responsible for a reduced enzymatic activity below 10% of normal.^114^, ^115^

## 6 CONCLUSIONS

Hereditary ataxias are a group of inherited diseases that share similar clinical characteristics. They can be divided into cerebellar cortical degenerations, spinocerebellar degenerations, cerebellar ataxias without substantial neurodegeneration, and multifocal degenerations with predominant (spino)cerebellar component. These diseases have different underlying causes and pathogenic mechanisms, but have comparable clinical phenotypes. Most of these diseases appear to be caused by variants interfering with basic cellular functions as autophagy and degradation, cation trafficking, or transport (Table 1), which explains why they have such extensive consequences on the CNS of these dogs. This review aims to provide an overview of currently described hereditary ataxia syndromes. As new diseases and genetic variants are constantly being recognized and discovered, this classification proposal will likely need to evolve and be updated in time, as our understanding of diseases continuously progresses.

## CONFLICT OF INTEREST DECLARATION

Authors declare no conflict of interest.

## OFF‐LABEL ANTIMICROBIAL DECLARATION

Authors declare no off‐label use of antimicrobials.

## INSTITUTIONAL ANIMAL CARE AND USE COMMITTEE (IACUC) OR OTHER APPROVAL DECLARATION

Authors declare no IACUC or other approval was needed.

## HUMAN ETHICS APPROVAL DECLARATION

Authors declare human ethics approval was not needed for this study.

## Supporting information


**File S1.** Details of the specific variants associated with the breed‐specific diseases discussed in Table 1.

**Movie S1.** Dancing bouncing gait (spinocerebellar ataxia) in a 1.5 years old Jack Russell Terrier with SAMS.

**Movie S2.** Myokymia in a 5 months old Malinois Shepherd dog with SAMS.

**Movie S3.** Neuromyotonia in a 2 years old Jack Russell Terrier with SAMS.

**Movie S4.** SAMS phenotype in a 4 months old Bouvier des Ardennes, showing a wide‐based stance, truncal sway, and clear hypermetric and dysmetric generalized ataxia, worse on the hind limbs.

**Movie S5.** Severe cerebellar phenotype (SDCA1) in a 7‐week‐old Bouvier des Ardennes, showing a severe generalized borderline ambulatory hypermetric ataxia with pronounced truncal sway. The second puppy is the same dog as in Movie S4, no clear ataxia is present at 7 weeks of age.

**Movie S6.** SAMS‐like syndrome in a 1‐year‐old Malinois Shepherd Dog, showing severe generalized hypermetric ataxia (worse on the hindlimbs) associated with a mild to moderate degree of paraparesis and a mild palmigrade stance.

**Movie S7.** Hereditary ataxia in a 2.5 years old Australian Shepherd, showing a clear dancing bouncing gait.

**Movie S8.** NCL4A in a 6 years old American Staffordshire Terrier, showing a wide‐based stance, bilateral vestibular ataxia, and clear hypermetric gait.

## Data Availability

Some or all data generated or analyzed during this study are included in this published article or in the data repositories listed in References.
