# Supplementary material for: Phenotypic and genetic aspects of hereditary ataxia in dogs
Source: J Vet Intern Med. 2023 Jun 21;37(4):1306–22. doi: 10.1111/jvim.16742 (PMC10365067; doi:10.1111/jvim.16742)
Supplement: Supplementary file 1 — File S1. Details of the specific variants associated with the breed‐specific diseases discussed in Table 1. [file JVIM-37-1306-s002.pdf]

Supplementary Information File 1. Overview of the described canine variants.

OMIA ID[1]	Gene	Gene symbol	Variant	Breed(s)	Reference	Phenotype MIM ID[2]
**Cerebellar cortical degenerations**						
002092-9615	483706	*SPTBN2*	XM_038424853.1:c.5855_5862del (p.(Ile1952Argfs*28))	Beagle	Forman et al., 2012[6]	600224,615386
001692-9615	480409	*SEL1L*	XM_038674398.1:c.1972T>C (p.(Ser658Pro))	Finnish Hound	Kyöstilä et al., 2012[7]	NA
001913-9615	479277	*RAB24*	XM_038663268.1:c.113A>C (p.(Gln38Pro))	Gordon Setter, Old English Sheepdog	Agler et al., 2014[8]	NA
002034-9615	474985	*SNX14*	XM_038684085.1:c.2713+1G>A	Hungarian Vizsla	Fenn et al., 2016[9]	616354
**Spinocerebellar Degenerations**						
001820-9615	483745	*CAPN1*	XM_038425033.1:c.344G>A (p.(Cys115Tyr))	PRT, JRT	Forman et al., 2013[13]	616907
002089-9615	488635	*KCNJ10*	XM_038448705.1:c.627C>G (p.(Ile209Met))	JRT, PRT, Patterdale Terrier, SHFT, Toy Fox Terrier.	Gilliam et al., 2014[14]	612780
002089-9615	488635	*KCNJ10*	XM_038448705.1:c.986T>C (p.(Leu329Pro))	Belgian Malinois Shepherd, Bouvier des Ardennes	Van Poucke et al., 2017[18]	612780
002279-9615	478239	*SLC12A6*	XM_038441820.1:c.178_181delinsCATCTCACTCAT (p.(Met60Hisfs*14))	Belgian Malinois Shepherd	Van Poucke et al., 2019[20]	218000
002194-9615	477604	*SCN8A*	XM_038438133.1:c.4898G>T (p.(Gly1633Val))	Alpine Dachsbracke	Letko et al., 2019[22]	600702,614306,614558,617080
**Cerebellar ataxia without substantial neurodegeneration**						
000078-9615	484024	*GRM1*	XM_038654394.1:c.2331_2332insN[62]	Coton de Tulear	Zeng et al., 2011[23]	614831,617691
002097-9615	476548	*ITPR1*	NC_051824.1:g.12923647CTT[7_651]	Italian Spinone	Forman et al., 2015[24]	117360,206700,606658
002240-9615	479129	*KCNIP4*	XM_038662081.1: c.487T>C (p.(Trp163Arg))	Norwegian Buhund	Jenkins et al., 2020[25]	NA
**Multifocal degenerations with predominant (spino)cerebellar component**						
NA	476255	*SERAC1*	XM_038654522.1:c.1536G>A (p.(Trp512*))	Kerry Blue Terrier	Guo et al., 2013[26]	614739
NA	476255	*SERAC1*	XM_038654522.1:c.182+1_182+4del	Chinese Crested		614739
002110-9615	489479	*ATP1B2*	XM_038665439.1:c.130_131ins[LT796559.1:g.50-276]	Belgian Malinois Shepherd	Mauri et al., 2017[27]	NA
002367-9615	479346	*SELENOP*	NC_051808.1: g.67456991_67473571del	Belgian Malinois Shepherd	Christen et al., 2021[28]	601484

000827-9615	612123	*PNPLA8*	XM_038423736.1:c.1169_1170dup (p.(His391Phefs*4))	Australian Shepherd	Abitbol et al., 2022[29]	251950
002522-9615	610876	*HACE1*	XM_038684251.1:c.1001del (p.(Gly334Valfs*34))	Black Norwegian Elkhound	Bellamy et al., 2022[30]	616756
002294-9615	603667	*SLC25A12*	XM_038447060.1:c.1370C>T (p.(Pro457Leu))	Nova Scotia Duck Tolling Retriever	Christen et al., 2022[31]	612949
001954-9615	484953	*ATG4D*	XM_038428641.1:c.1372G>A (p.(Ala458Thr))	Lagotto Romagnolo	Kyöstilä et al., 2015[32]	NA
002152-9615	489374	*VPS11*	XM_038664676.1:c.2504A>G (p.(His835Arg))	Rottweiler	Lucot et al., 2018[33]	616683
001503-9615	480460	*ARSG*	XM_038674991.1:c.296G>A (p.(Arg99His))	American & Pitbull Staffordshire	Abitol et al., 2010[34]	618144
001505-9615	483662	*CTSD*	NM_001025621.1:c.597G>A (p.(Met199Ile))	American Bulldog	Awano et al., 2006[35]	116840
000402-9615	403873	*GLB1*	NM_001037641.1:c.179G>A (p.(Arg60His))	Portuguese Water	Wang et al., 2000[36]	230500,230600,230650,253010

[1] Online Mendelian Inheritance in Animals, OMIA. Faculty of Veterinary Science, University of Sydney, Sydney. URL: http://omia.angis.org.au/ (accessed January 2021).
[2] Online Mendelian Inheritance in Man, OMIM. McKusick-Nathans Institute of Genetic Medicine, Johns Hopkins University, Baltimore, MD. URL: https://omim.org/ (accessed January 2021).
